# Disparities in the prevalence and risk factors of anaemia among children aged 6–24 months and 25–59 months in Ethiopia

**DOI:** 10.1017/jns.2020.29

**Published:** 2020-08-27

**Authors:** Tafere Gebreegziabher, Nigatu Regassa, Micaela Wakefield, Kelly Pritchett, Susan Hawk

**Affiliations:** 1 Food Science and Nutrition, Department of Health Sciences, Central Washington University, Ellensburg, WA 98926-7571, USA; 2 College of Pharmacy and Nutrition, University of Saskatchewan, Saskatoon, SK, Canada

**Keywords:** Anaemia, Children, Ethiopia, Risk factors

## Abstract

Despite global efforts made to address anaemia, the prevalence remains high in most Sub-Saharan African countries. In Ethiopia, anaemia poses a very strong public health concern. The purpose of the present study was to examine the key risk factors related to anaemia among children aged 6–24 months (younger age group) and 25–59 months (older age group). We used the 2016 Ethiopian Demographic and Health Survey data, collected from 11 023 mothers with under five children. Ordered logistic regression modelling was used for assessing risk factors of childhood anaemia. The results suggest that the prevalence of anaemia was 72 % in the younger and 49 % in the older age groups. The risk factors for anaemia in the younger age group were morbidity (odds ratio (OR) 1⋅77; CI 1⋅21, 2⋅60), having no piped water source (OR 1⋅76; CI 1⋅07, 3⋅01) and no toilet facility (OR 1⋅60; CI 1⋅07, 2⋅38). The key risk factors for anaemia in the older age group were no micronutrient intake (OR 1⋅69; CI 1⋅23, 2⋅31), having a young mother (15–24 years old) (OR 1⋅35; CI 0⋅84, 1⋅91) and a non-working mother (OR 1⋅50; CI 1⋅15, 1⋅96). Anaemia also varied by region, place of residence and economic factors. Multiple factors contributed to the high prevalence of anaemia. Given the structural problem that the country has intervention strategies should consider the unique characteristics of regions and rural residences where the prevalence of anaemia is above the national average.

## Introduction

Globally, children under the age of five are most severely impacted by anaemia when compared to other population groups^(1)^. Despite measures to address this health concert, anaemia continues to persist as one of the most prevalent public health concerns in several African and Asian countries. In 1995, the global prevalence of anaemia in children under the age of five soared at 47 %; yet, in 2011, the prevalence only decreased by 4 %. In East Africa, it reached a staggering 55 %^(1)^, while in Ethiopia, the prevalence of anaemia in young children was estimated to be as high as 72 %^(2)^. In a recent meta-analysis, anaemia in school-age children nationally estimated to be 23 %. It is important to note that this value fluctuates regionally, and in some locations, it can be upward of 44 %^(3)^.

Of paramount concern is the lasting effect of iron deficiency anaemia on childhood development. A chronic deficit can delay or inhibit a child's full potential and negatively impact lifelong development^(4)^. Not only does anaemia impact people on an intrapersonal level, but it can also detrimentally impact social and economic realms^(5)^. Increased mortality is a dire consequence of anaemia in young children^(6)^. While anaemia mortality in some cases is only associated with severe anaemia^(1)^, a review from six African countries reveals the vulnerability of young children to the lethal consequence of this nutrient deficiency. Here, children aged 28 d to 5 years were followed. Results indicated that for every 10 g/l increase in haemoglobin concentration among mild and moderately anaemic children, the risk of death decreased by 24 %. Thus, nearly 1⋅8 million deaths could be prevented annually with an improvement in iron status^(7)^.

The World Health Organization defined anaemia in children age 6–59 months as having a haemoglobin concentration below 110 g/l^(8)^. Of importance is to note that iron deficiency is only one of the contributing factors to this global health problem. Approximately 50 % of anaemia cases worldwide are linked to iron deficiency. Other contributing factors to anaemia may include deficiencies in vitamin B12, folate or vitamin A, genetic disorders, parasitic infections and chronic inflammation. These may be further exacerbated by low socio-economic status, poor education, poor sanitation and hygiene^(4,9)^.

Young children under 5 years of age are particularly susceptible to anaemia morbidity and mortality, particularly in low-income countries^(10)^. The underlying cause of the anaemia may be multifactorial. Micronutrient deficiencies, especially iron, zinc and vitamin A are widespread in many low-income countries including Ethiopia. Low dietary diversity, driven by food insecurity and lack of knowledge, is one driving force behind the deficiencies^(11–14)^. Poor sanitation, parasitic infection and overall poor nutritional status are also contributing factors to the alarming prevalence of anaemia in young children^(15)^. In conjunction, all of these factors weaken the immune system and may thereby increase susceptibility to infection and mortality^(15,16)^.

The first 1000 d of life – the period from conception to 2 years of age – is the most critical period for growth and development. For example, iron and zinc deficiency during early childhood can result in a life prone to infections and further impair cognitive and physical processes^(17,18)^. Surprisingly, most studies do not subdivide children by age when examining the prevalence of anaemia. Given the specific physiological and nutritional needs during early development, separating children into age groups is critical. Children grow and change dramatically over the course of their first 5 years of life. By grouping children into smaller subcategories, it ensures the understanding of the prevalence of and associations with anaemia among those different age groups.

**Fig. 1.**
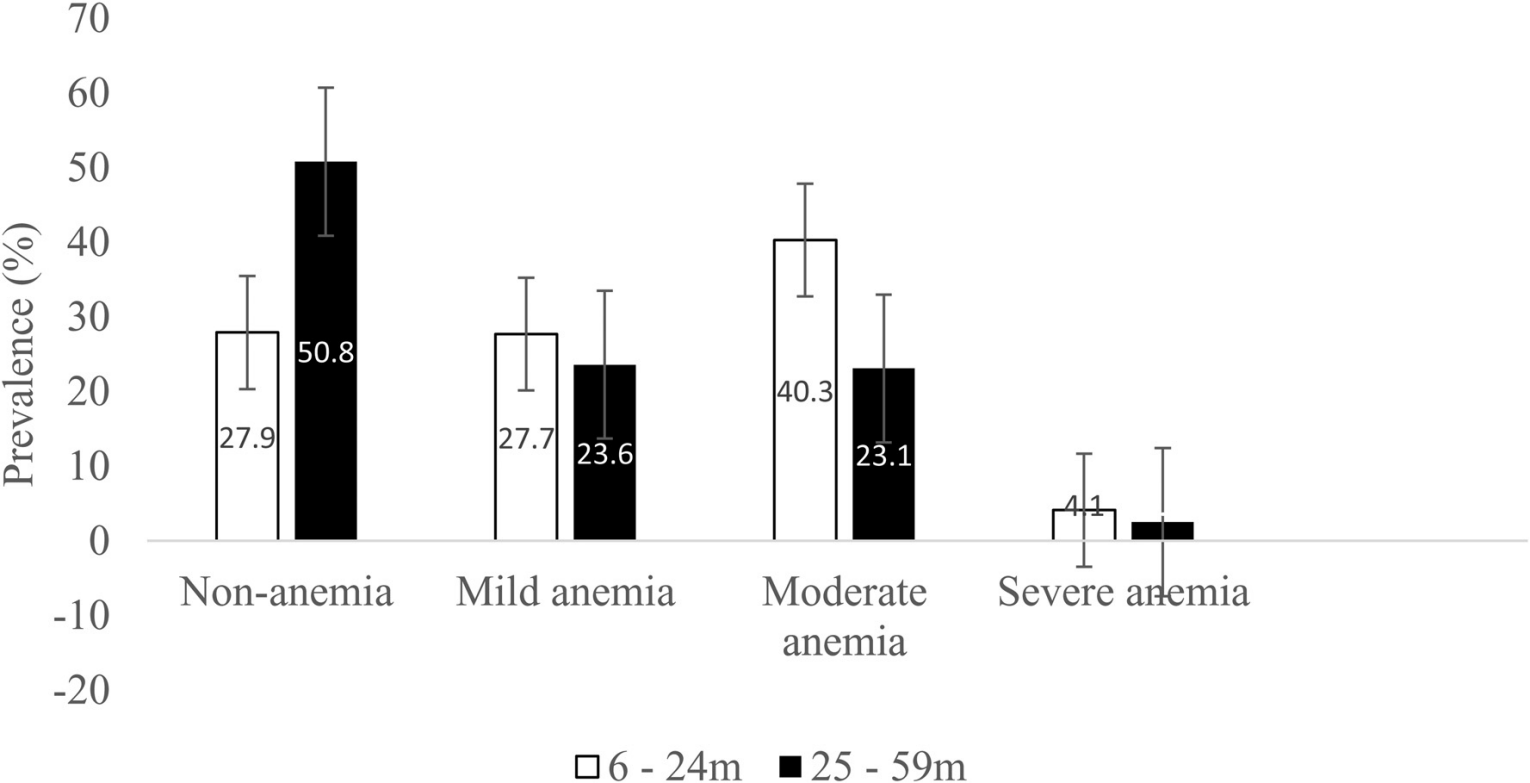
Prevalence of anaemia among children aged 6–24 and 25–59 months (*n* 8476).

In the present study, we aimed to examine the key predictors of anaemia among children aged 6–24 months and those 25–59 months (Fig. 1). To the best of our knowledge, the present study is the first of its kind in Ethiopia addressing a separate analysis of risk factors of anaemia for the two age groups.

## Data source and methodology

### Data source, study design and study population

The present study utilised data from the Ethiopian Demographic and Health Survey (EDHS 2016) data. The EDHS is a multistage complex cross-sectional survey^(2)^. The specific data were collected from 645 enumeration areas (EAs) that were randomly selected from all administrative divisions of the country. The selection of households within the selected EAs was *via* systematic random sampling^(2)^. The present study focused exclusively on what is deemed the kid's file. This data set included a wide range of health and socio-demographic information about mothers and their children under the age of five. The total sample size considered for the present analysis was 9794 children age 6–59 months. Infants below 6 months of age were excluded given that EDHS did not measure haemoglobin level for this age bracket. The detailed description of methods, design, instruments, participants and sampling frame was previously published by the DHS program at: https://dhsprogram.com/publications/publication-fr328-dhs-final-reports.cfm.

The EDHS followed internationally accepted standard protocols, data collection tools and procedures. Participation in the survey was voluntary. DHS granted public access to the microdata.

### Outcome and exposure variables

The outcome variable was defined by haemoglobin (Hgb) level <11 g/dl^(8)^. The haemoglobin was measured using a HemoCue®201 analyzer which was adjusted for altitude^(2)^. The coding for the outcome variable ranged from 1 to 4: (1) non-anaemia (Hgb level ≥ 11 g/dl); (2) mild anaemia (Hgb level 10–10⋅9 g/dl); (3) moderate (Hgb level 7–9⋅9 g/dl) and (4) severe (Hgb level <7 g/dl). Two children under age five become anaemic and, thus, were included in the analysis.

We divided the exposure variables into four major categories: child characteristics (sex of the child, morbidity status, micronutrient intake, deworming, child diet diversity, breast feeding, stunting and wasting); maternal characteristics (maternal education, maternal age, maternal work status, maternal haemoglobin, mother's age at first birth, mother's height, BMI and maternal health service utilisation); household factors (household size, religion, wealth index, access to toilet facility and sources of drinking water). The community-level variables were regions and place of residence.

Morbidity status was measured based on reported signs and symptoms for diarrhoea and acute respiratory infection. These included cough, fever and congestion around the chest during a reference period of 2 weeks prior to the survey date. A child's size at birth was categorised as small, average and large. For micronutrient intake and deworming, the EDHS collected information if the child was given a micronutrient and deworming supplement during a reference period of 6 months prior to the survey date. A mother's age was classified into three groups: 15–24; 25–34; 35–49. Maternal work status was measured as ‘yes/no’ for a reference period of 12 weeks prior to the survey date. Household income was approximated by a self-reported asset-based household wealth index^(2)^. All remaining variables were used in their original coded format by EDHS.

Fifteen exposure variables were initially selected for the present study. The inclusion of these variables was considered our literature review, research questions/hypothesis, correlations among variables and amount of missing values in each individual variable.

### Statistical analysis

Data cleaning, management and analysis were carried out using IBM Statistics SPSS version 23. In order to capture the key predictors of childhood anaemia, a wide range of explanatory variables were analysed for their significance in the bivariate/unconditional ordered logistic regression analysis (not shown). All variables having *P* < 0⋅20 significance level at this stage were further included in a multiple ordered logistic regression model^(19)^. Multicollinearity among the explanatory variables was tested using the variance inflation factor (VIF) and those with VIF greater than 2⋅5 were removed from the analysis^(20)^.

Ordered logit was used for assessing the key risk factors of childhood anaemia, controlling for all possible confounders. The ordered logit inputs parallel regressions assumptions where all beta values of the various subequations are the same except at their intercept. In total, twelve variables were entered in the multiple ordered logistic regression model to see the adjusted effects of each potential predictor. Odds ratios (ORs) with 95 % confidence intervals (CIs) were calculated for each factor in the cumulative logistic regression model.

A sampling weight variable computed by Central Statistical Agency (CSA) and Inner City Fund (ICF) International was used in all analyses to ensure that the final estimates were representative of the national population. A robust standard error of regression coefficient was used to better address the stratification and clustering effects which is a common issue in cross-sectional designs.

## Results

### Characteristics of respondents

Table 1 displays the characteristics of children aged 6–24 months and 25–59 months, giving a weighted sample of 9794 children. With respect to the child's weight at birth, most were average in size, 40 and 43 % for younger and older children, respectively. Small-sized children accounted for 28 and 24 % of the two groups, respectively. Approximately 90 % of the children representing both groups resided in households with poor water access (i.e. no piped water). Similarly, a nearly equal proportion of children in both groups lived in households with no toilet facilities. Close to 90 % of the children were living in rural households, and the proportion of those living in poor/poorer household's amount close to 50 %. One-third of children had mothers with anaemia. More than 50 % of children were reported to have been living in a household size of 4–6 members. Most of the mothers were in the age group of 25–34 years. For both groups, the proportions of mothers with no education were high, 62 % in the younger and 69 % in the older age group.

**Table 1.** Socio-economic and demographic characteristics of households by children's age group (*n* 9794), EDHS 2016, Ethiopia

Variables	6–24 months *n* (%)	25–59 months *n* (%)
Sex of child		
Male	1664 (48⋅1)	3451 (54⋅5)
Female	1793 (51⋅9)	2886 (45⋅5)
Size of child at birth		
Small	948 (27⋅7)	1532 (24⋅3)
Average	1367 (39⋅9)	2696 (42⋅8)
Large	1112 (32⋅5)	2069 (32⋅9)
Source of drinking water for the household		
No piped	3031 (89⋅7)	5739 (91⋅6)
Piped	348 (10⋅3)	530 (8⋅4)
Toilet facility		
No facility	1260 (37⋅3)	2420 (38⋅7)
There is facility	2118 (62⋅7)	3837 (61⋅3)
Place of residence		
Rural	3044 (88⋅1)	5681 (89⋅6)
Urban	413 (11⋅9)	656 (10⋅4)
Maternal anaemia		
Not anaemic	2272 (67⋅9)	4097 (67⋅0)
Anaemic	1072 (32⋅1)	2022 (33⋅0)
Household wealth index		
Poor/poorer	1543 (44⋅6)	3023 (47⋅7)
Middle	738 (21⋅4)	1316 (20⋅8)
Rich/richer	1176 (34)	1997 (31⋅5)
Household size		
1–3	448 (13⋅0)	579 (9⋅1)
4–6	1770 (51⋅2)	3213 (50⋅7)
≥7	1239 (35⋅8)	2545 (40⋅2)
Maternal age (years)		
15–24	943 (27⋅3)	1096 (17⋅3)
25–34	1798 (52⋅0)	3447 (54⋅4)
35–49	716 (20⋅7)	1794 (28⋅3)
Maternal education		
Illiterate	2143 (62⋅0)	4418 (69⋅7)
Literate	1314 (38⋅0)	1919 (30⋅3)

### Risk factors of childhood anaemia

Table 2 presents the results of ordered logistic regression for key determinants of anaemia among the younger and older age groups. Generally, a positive coefficient (OR > 1) means that increases in the explanatory variable lead to higher values of the response variable, while a negative coefficient (OR < 1) means that increases in the explanatory value lead to a decreased in the response value. Seven variables appeared to be associated with anaemia among the younger age group (sex of the child, household size, childhood morbidity, source of drinking water, access to toilet facility, deworming and birth weight). In the older age group, six variables predicted anaemia, including micronutrient intake, deworming, household size, maternal work status, maternal age and birth weight. Three significant detectors were shared among groups. These included deworming, size of child at birth and household size.

**Table 2.** Ordinal logistic regression for selected predictors and level of anaemia among under five children, Ethiopia (Odds ratios and 95% confidence intervals)

Variables	Children 6–24 months (*n* 3102)		Children 25–59 months (*n* 5381)	
	OR (95 % CI)	*P* value	OR (95 % CI)	*P* value
Sex of child				
Male	0⋅52 (0⋅38, 0⋅72)	**<0⋅001**	0⋅78 (0⋅57, 1⋅08)	0⋅14
Female	1		1	
Morbidity*				
No	1		1	
Yes	1⋅77 (1⋅21, 2⋅60)	0⋅003	1⋅34 (0⋅98, 1⋅84)	0⋅068
Source of drinking water				
No piped	1⋅76 (1⋅07, 3⋅01)	**0⋅02**	1⋅33 (0⋅96, 1⋅85)	0⋅08
Piped	1		1	
Micronutrient intake^†^				
No	0⋅93 (0⋅78, 1⋅12)	0⋅46	1⋅69 (1⋅23, 2⋅31)	**<0⋅001**
Yes	1		1	
Deworming^‡^				
No	1⋅71 (1⋅34, 2⋅17)	**<0⋅001**	1⋅50 (1⋅19, 1⋅89)	**0⋅005**
Yes	1		1	
Toilet facility				
No facility	1⋅60 (1⋅073, 2⋅38)	**0⋅02**	0⋅75 (0⋅51, 1⋅11)	0⋅14
There is facility	1		1	
Mother working				
No	0⋅97 (0⋅67, 1⋅41)	0⋅87	1⋅50 (1⋅15, 1⋅96)	**0⋅003**
Yes	1		1	
Size of child at birth				
Small	1⋅60 (1⋅07, 2⋅38)	**0⋅02**	2⋅04 (1⋅28, 3⋅27)	**0⋅003**
Average	1⋅43 (0⋅91, 2⋅24)	1⋅12	1⋅06 (0⋅70, 1⋅61)	0⋅77
Large	1		1	
Received MCHC^§^				
No	0⋅98 (0⋅83, 1⋅15)	0⋅805	0⋅78 (0⋅63, 0⋅85)	0⋅79
Yes	1		1	
Dietary diversity score^||^	0⋅997 (0⋅95, 1⋅05)	0⋅911	1⋅01 (0⋅95, 1⋅069)	0⋅771
Household size				
1–3	0⋅76 (0⋅52, 1⋅13)	**0⋅02**	0⋅48 (0⋅29, 0⋅78)	**0⋅003**
4–6	0⋅84 (0⋅76, 1⋅08)	**0⋅041**	0⋅61 (0⋅38, 0⋅95)	**0⋅031**
≥7	1		1	
Maternal age (years)				
15–24	0⋅70 (0⋅42, 1⋅15)	0⋅16	1⋅35 (0⋅84. 1⋅91)	**0⋅044**
25–34	0⋅86 (0⋅58, 1⋅27)	0⋅44	1⋅22 (0⋅85, 1⋅75)	0⋅271
35–49	1		1	
Maternal education				
Illiterate	1⋅15 (0⋅68, 2⋅01)	0⋅613	1⋅16 (0⋅65, 2⋅07)	0⋅63
Literate	1			

OR, odds ratio.

* Child had one or more of the listed health problems in the past 2 weeks (diarrhoea, fever, cough or shortness of breath).

† Child has taken one or more of the listed micronutrients (Iron, zinc or vitamin A).

‡ Child received drugs for the intestinal parasite in the last 6 months.

§ Maternal and child health care (computed from prenatal, antenatal and postnatal care).

|| Dietary diversity score (continuous).

In the younger age group, male children are 48 % less likely (OR 0⋅52; CI 0⋅38, 0⋅72) to be in one of the higher categories as they are to be in the lowest category. Children living in small (0–3) and medium (4–6) sized households were 24 % (OR 0⋅76; CI 0⋅52, 1⋅13) and 16 % (OR 0⋅84; CI 0⋅76, 1⋅08) less likely to fall in the higher anaemia category as they are to be in the lowest category. In the older age group, the likelihood of falling in a higher anaemia level decreased by 52 % for children living in smaller-sized households (OR 0⋅48; CI 0⋅29, 0⋅78).

In the younger age group, children who had an illness during the reference period were 1⋅77 times (CI 1⋅21, 2⋅60) more likely to be in one of the higher categories compared to being in the lowest category. Children living in households without piped water source had 1⋅76 times (CI 1⋅07, 3⋅01) chance of falling in the higher anaemia category than being in the lower category. Those who did not receive deworming tablets had a higher chance of experiencing the higher-order anaemia level (OR 1⋅71; CI 1⋅34, 2⋅17) than falling into the lower category. Having no access to a toilet facility was also found to be significantly associated with childhood anaemia. Those living in households with no toilet facility had 1⋅60 times (CI 1⋅073, 2⋅38) more chance to fall in the higher anaemia category than being in the lower category. It is also noted that children having small size birth weight were 1⋅60 times (CI 1⋅07, 2⋅38) more likely to fall in the higher anaemia category than the lower category. In the older age group, the odds of falling in the higher anaemia category, than being in the lower category, were 1⋅69 times (CI 1⋅23, 2⋅31) higher for children who did not take micronutrient supplement during the reference period. Similarly, children who did not take deworming tablets had a higher chance of being in the higher anaemia category (OR 1⋅50; CI 1⋅19, 1⋅89) than being in the lower category. The odds of being in a higher anaemia level were higher for a non-working mother by 1⋅50 (CI 1⋅15, 1⋅96), for children of smaller-sized birth weight by 2⋅04 times (CI 1⋅28, 3⋅27) and for younger mothers (15–24 years old) by 1⋅35 times (CI 0⋅84, 1⋅91) compared to their respective reference categories.

Table 3 presents the results of ordered logistic regression for key social factors hypothesized to predict anaemia. All the three factors appeared to predict anaemia. The odds of being in the higher anaemia category were 2⋅33 times for the younger age group and 1⋅66 times for the older age group for children residing in Afar region. In Somali region, the odds of being in the higher anaemia category were 2⋅73 times for the younger age group and 2⋅84 times for the older age group. The odds of being in the higher anaemia level, as they are to be in the lowest category, were higher by 2⋅06 times in Benishangul Gumuz region (CI 1⋅39, 3⋅07) in the younger age group and 1⋅81 times in Dire Dawa (CI 1⋅16, 2⋅82) in the older age group.

**Table 3.** Correlates of anaemia among children aged 6–24 months and 25–59 months: social factors, Ethiopia 2016

Variables	6–24 months (*n* 3102)	25–59 months (*n* 5381)
	OR (95 % CI)	OR (95 % CI)
Regions		
Addis Ababa^a^	Ref.	Ref.
Tigray	1⋅20 (0⋅74, 1⋅95)	0⋅76 (0⋅21, 1⋅13)
Afar	2⋅33 (1⋅29, 4⋅20)**	1⋅66 (1⋅09, 2⋅53)*
Amhara	0⋅73 (0⋅45, 1⋅20)	0⋅46 (0⋅31, 0⋅69)***
Oromia	1⋅03 (0⋅63, 1⋅69)	1⋅08 (0⋅73, 1⋅60)***
Somali	2⋅73 (1⋅55, 4⋅78)***	2⋅84 (1⋅88, 4⋅27)***
Benishangul	2⋅06 (1⋅39, 3⋅07)***	0⋅98 (0⋅71, 1⋅25)
SNNPR	0⋅52 (0⋅32, 0⋅86)*	0⋅70 (0⋅47, 1⋅05)
Gambela	1⋅04 (0⋅60, 1⋅80)	0⋅97 (0⋅63, 1⋅49)
Harari	1⋅48 (0⋅83, 2⋅66)	1⋅18 (0⋅76, 1⋅84)
Dire Dawa	1⋅81 (0⋅99, 3⋅31)	1⋅81 (1⋅16, 2⋅82)**
Place of residence		
Rural	Ref.	Ref.
Urban	0⋅71 (0⋅54, 0⋅93)*	0⋅76 (0⋅61, 0⋅94)*
Religion		
Orthodox	Ref.	Ref.
Catholic	0⋅80 (0⋅34, 1⋅86)	0⋅99 (0⋅56, 1⋅76)
Protestant	0⋅79 (0⋅60, 1⋅06)	1⋅01 (0⋅83, 1⋅23)
Muslim	0⋅70 (0⋅55, 0⋅90)**	0⋅69 (0⋅58, 0⋅82)***
Traditional	0⋅35 (0⋅15, 0⋅81)*	0⋅47 (0⋅26, 0⋅86)*
Other	1⋅36 (0⋅60, 3⋅10)	0⋅45 (0⋅25, 0⋅84)*

OR, odds ratio; Ref., reference category; SNNPR, South Nations, Nationalities and Peoples' Region.

* *P* < 0⋅05, **P < 0⋅01, ***P < 0⋅001.

a Capital city.

In both groups, the odds of being in the higher anaemia level decreased by 29 % (OR 0⋅71; CI 0⋅54, 0⋅93) and 24 % (OR 0⋅76; CI 0⋅61, 0⋅94) among children residing in urban areas. In terms of religion, children from Muslim families had a lower chance of being in the higher anaemia level, than being in the lower category, by 30 and 31 % for the two age groups, respectively. Similarly, children with parents following traditional religion had a lower likelihood of being in the higher category as they are to be in the lowest category.

## Discussion

The present study examined key predictors and prevalence of childhood anaemia in Ethiopia based on nationally representative data. The weighted prevalence computed in this analysis was 53 % for the total study sample (age 6–59 months) and 72 % for the younger age group. These figures are unacceptably high and demonstrate the urgency that should be taken to address this epidemic. In a recent study based on national data from Sub-Saharan African countries, the average regional prevalence was 59⋅9 %, ranging from 23⋅7 % in Rwanda to 87⋅9 % in Burkina Faso^(21)^.

Our analysis reveals that a combination of child, maternal, household and community-level factors are contributing factors for the high prevalence of childhood anaemia in Ethiopia. The prevalence of anaemia was significantly different among regions, religious groups and area of residence. Living in certain regions and in rural areas, as well as being a member of some religious groups were strongly associated with an increased likelihood of having childhood anaemia. This could be due to differences in access to resources, level of education, cultural and dietary practices, as well as disparities in income level^(22)^. Individuals living in urban areas may be more educated, have higher income and have better access to resources compared to rural areas which are directly or indirectly related to poverty. Population distribution of religious groups is also different by region and by area of residence. This may contribute to the difference in the burden of anaemia by religion. Specifically, it may be attributable to differences in dietary patterns among different religions. In India, anaemia was least prevalent among children from Muslim households compared to Christian and Hindu households. This difference was attributed to religion-based dietary differences^(23)^.

While the prevalence of anaemia was different among the two age groups, so were the contributing factors. In the younger age group, morbidity appeared to be a significant predictor of anaemia. However, in the present analysis, the three most commonly used indicators of illness were considered (diarrhoea, acute respiratory infections and fever). Consistent with these findings, a study conducted in rural Indonesia validated that present diarrhoea or a history of diarrhoea within the previous week were associated with anaemia in children under age five after controlling for other factors that lead to diarrhoea and anaemia^(24)^. Though the present analysis did not consider malaria as a predictor in the logistic regression model, malaria is one of the most commonly reported risk factors for anaemia in the tropics^(25,26)^. A study conducted in Cameroon reported that malaria was a major cause of anaemia in children under 5 years^(27)^. Furthermore, approximately 68 % of the population are at risk for malaria in Ethiopia^(28)^.

Concomitant infection with malaria and intestinal parasites also increases susceptibility to other infections and are also associated with anaemia^(29,30)^. Similarly, the present study suggested that not taking drugs for intestinal parasites was associated with anaemia in both age groups. Intestinal parasitic infections cause blood and iron loss through the intestinal tract and subsequent destruction of erythrocytes resulting in decreased haemoglobin levels^(31,32)^.

Poor sanitation and hygiene problems in Ethiopia, particularly in the rural areas is widespread. In the present study, more than 90 % of the children drank from unprotected water sources, 38 % had no toilet facilities which were both associated with anaemia in the younger age groups. Poor personal hygiene was associated with hookworm infestation as well as anaemia in young children in Ethiopia^(33)^. No access to protected water and occasional interruption of piped water in Addis Ababa (the capital city of Ethiopia) slums was a cause of *Escherichia coli* infection and acute diarrhoea in over 83 % of under 5 years old children. The primary reason for this infection was unhygienic handling and utilisation of stored water^(34)^.

Children (age 25–59 months) who reported not taking micronutrient supplements were likely to be anaemic. Although the amount and frequency of intake were not known, the types of nutrients included in this analysis were iron, zinc and vitamin A. Iron needs at a younger age is high. Iron deficiency anaemia, particularly microcytic anaemia, is the most common form of anaemia in children, which contributes to 50 % of all cases of anaemia^(9,35)^. Not only anaemia but also iron deficiency can cause delays in psychomotor and cognitive development in young children^(36)^. As a result, it is recommended that iron intake be increased through fortification, supplementation and dietary diversification in order to meet iron needs^(37)^. Zinc is one of the most common micronutrient deficiencies in young children other than iron and vitamin A^(11)^. Zinc may affect haemoglobin production through zinc-dependent enzymes involved in haemoglobin synthesis^(38)^. Nevertheless, evidence shows that a combination of vitamin A and zinc in addition to iron has a better effect in increasing haemoglobin levels than iron alone^(39)^. This synergistic role between zinc and vitamin A is important in decreasing the prevalence of anaemia^(39)^. A study in pre-school children in China showed that supplementation with multiple micronutrients was more effective in reducing the prevalence of anaemia than with a single micronutrient supplement^(40)^.

Increasing nutrient intake through supplementation and fortification may decrease micronutrient deficiencies in children. However, supplementation should follow deworming. Unless parasitic infestation or other infectious diseases are treated, micronutrient supplementation and fortification may aggravate morbidity. In a double-blind randomised trial in 6-month-old anaemic infants in Kenya consuming home-fortified maize with micronutrient powder that included 12⋅5 or 2⋅5 mg iron daily for 4 months, adversely affected the gut microbiome, increased pathogen abundance and caused intestinal inflammation^(41)^. Similar results were found in another double-blind randomised study in non-anaemic or mildly anaemic Kenyan infants^(42)^. In another study, iron-fortified infant formula significantly increased diarrhoea incidence in infants and children^(43)^. In the present analysis, those children who did not receive medication for intestinal parasites were more likely to be anaemic than those undergoing treatment. Evidence suggested that the recently implemented deworming programme in school children in Ethiopia shows promising results in decreasing parasitic infestation and prevalence of anaemia^(44,45)^.

Our findings suggest that, for both age groups, the likelihood of being anaemic is higher among low birth weight (LBW) children. Haemoglobin concentration of newborn babies normally falls to a minimal level until age 12 weeks. In LBW and very low birth weight children, haematocrit volume decreases to approximately 24 %^(46,47)^. The reduction in haemoglobin and haematocrit is amplified in extremely LBW babies and is referred to as anaemia of prematurity^(47)^. Moreover, iron deficiency anaemia is the most common anaemia observed in young and LBW children because of the increased iron needs during their rapid growth spurt^(48)^. The condition is critical in countries like Ethiopia where complementary food is poorly diversified and inadequate^(49,50)^. A study showed that in some parts of Ethiopia, the majority of infants and young children consume very low dietary diversity only from 0 to 2 food groups. Besides, micronutrient intake from complementary food was also below estimated needs^(51)^.

Other factors such as maternal anaemia, environmental factors, social and economic factors prior to or during pregnancy may lead to an LBW^(52–54)^. Generally, children with birth weights (<2500 g) are more susceptible to morbidity and mortality^(52,55)^.

The odds of anaemia were higher in children of young mothers (15–24 years). At a younger age, women may not be physiologically ready to provide the nutritional needs of their children. Moreover, it could be related to a lack of knowledge and experience in childcare and feeding practices. In some instances, particularly in rural areas of Ethiopia where women get married at a very young age, it could be related to a lack of resources, and hence, a lack of adequate food to meet nutrient requirements for themselves and their children^(56,57)^.

Economic factors contributed significantly to anaemia in the present study. Children of mothers who do not have an employment or income were more likely to be anaemic which is likely attributable to limited access to good nutrition and health care. A study conducted in China found that the income of parents and feeding practices were significant predictors of anaemia in children under 36 months old^(58)^. Household size, which usually linked with income and wealth, was also an important factor in the present study. Children from small and average households were less likely to be anaemic for both age groups, while larger household size with limited resources could be a major contributing factor for household food insecurity.

### Strengths and limitations

These findings were based on a representative sample of respondents covering the entire regions in the country and can, therefore, be generalised to the larger population in Ethiopia. Given the fact that studies examining childhood anaemia are scarce, the findings may also be useful for planning, targeting and monitoring and evaluating progress in this outcome at the national scale. However, the present study has some limitations. Due to its cross-sectional nature, it was not possible to draw a temporal relationship between the various exposures and childhood anaemia. The high prevalence of anaemia in the study population (about 72 % among the young age groups) resulted in a lower variability in some of the hypothesized variables and unable to see the significant association with the outcome variables. Also, the data were self-reported and, thus, could be affected by recall and misclassification biases.

## Conclusion and policy implications

In conclusion, our findings suggest that the prevalence of anaemia is unacceptably high in children under five and severe in the youngest age group. It is difficult to determine a single cause of anaemia given that multiple factors contribute to its occurrence. In the present analysis, morbidity, sanitation and hygiene-related factors contributed to the likelihood of childhood anaemia. Thus, strengthening both nutrition-sensitive (such as health education, WASH (water, sanitation and hygiene) and promotion of maternal knowledge) and nutrition-specific interventions (such as micronutrient supplementation and deworming) may decrease the consistently higher prevalence of anaemia in the country. Furthermore, more attention should be given to rural households and disadvantaged regions where the prevalence of anaemia is above the national average. Addressing the structural problem that the country has including poor access to healthcare facilities, empowering women and reducing poverty in these disadvantaged areas should be a priority concern for policy-makers and local administrators. Region and religion were significant predictors of anaemia in the present study which could imply that interventions should be region-specific and should consider the existing nature of each region.
